# Tracking the cellulolytic activity of *Clostridium thermocellum* biofilms

**DOI:** 10.1186/1754-6834-6-175

**Published:** 2013-11-29

**Authors:** Alexandru Dumitrache, Gideon M Wolfaardt, David Grant Allen, Steven N Liss, Lee R Lynd

**Affiliations:** 1 Department of Chemical Engineering and Applied Chemistry, University of Toronto, 200 College St., Toronto, ON M5S 3E1, Canada; 2 Department of Chemistry and Biology, Ryerson University, 350 Victoria St., Toronto, ON M5B 2K3, Canada; 3 Stellenbosch Institute for Advanced Study Wallenberg Research Centre at Stellenbosch University, Stellenbosch 7600, South Africa; 4 School of Environmental Studies and Department of Chemical Engineering, Queen’s University, 99 University Ave., Kingston, ON K7L 3N6, Canada; 5 Thayer School of Engineering, Department of Biological Sciences, Dartmouth College, Hanover, NH 03755, USA

**Keywords:** Biofilm kinetics, Cellulose conversion, CO_2_ production, *Clostridium thermocellum*

## Abstract

**Background:**

Microbial cellulose conversion by *Clostridium thermocellum* 27405 occurs predominantly through the activity of substrate-adherent bacteria organized in thin, primarily single cell-layered biofilms. The importance of cellulosic surface exposure to microbial hydrolysis has received little attention despite its implied impact on conversion kinetics.

**Results:**

We showed the spatial heterogeneity of fiber distribution in pure cellulosic sheets, which made direct measurements of biofilm colonization and surface penetration impossible. Therefore, we utilized on-line measurements of carbon dioxide (CO_2_) production in continuous-flow reactors, in conjunction with confocal imaging, to observe patterns of biofilm invasion and to indirectly estimate microbial accessibility to the substrate’s surface and the resulting limitations on conversion kinetics. A strong positive correlation was found between cellulose consumption and CO_2_ production (R^2^ = 0.996) and between surface area and maximum biofilm activity (R^2^ = 0.981). We observed an initial biofilm development rate (0.46 h^-1^, 0.34 h^-1^ and 0.33 h^-1^) on Whatman sheets (#1, #598 and #3, respectively) that stabilized when the accessible surface was maximally colonized. The results suggest that cellulose conversion kinetics is initially subject to a microbial limitation period where the substrate is in excess, followed by a substrate limitation period where cellular mass, in the form of biofilms, is not limiting. Accessible surface area acts as an important determinant of the respective lengths of these two distinct periods. At end-point fermentation, all sheets were digested predominantly under substrate accessibility limitations (e.g., up to 81% of total CO_2_ production for Whatman #1). Integration of CO_2_ production rates over time showed Whatman #3 underwent the fastest conversion efficiency under microbial limitation, suggestive of best biofilm penetration, while Whatman #1 exhibited the least recalcitrance and the faster degradation during the substrate limitation period.

**Conclusion:**

The results showed that the specific biofilm development rate of cellulolytic bacteria such as *C. thermocellum* has a notable effect on overall reactor kinetics during the period of microbial limitation, when ca. 20% of cellulose conversion occurs. The study further demonstrated the utility of on-line CO_2_ measurements as a method to assess biofilm development and substrate digestibility pertaining to microbial solubilization of cellulose, which is relevant when considering feedstock pre-treatment options.

## Background

The strictly anaerobic thermophile, *Clostriudium thermocellum* has been used as model cellulolytic bacterium in numerous studies. It forms distinctive thin, often monolayer biofilms on cellulose [1] that lack an extracellular polymeric matrix typically found in biofilms. Cell-bound cellulosomes [2] have been demonstrated to provide the main extracellular hydrolytic activity on solid substrates, and up to 86% of oligomeric hydrolysis products are captured by the adherent bacterial population [1]. Upon further intracellular breakdown, the soluble sugars are processed through the Embden-Meyerhof pathway to a pyruvate intermediate, which is then predominantly converted to acetic acid and ethanol end-products with the stoichiometric co-production of carbon dioxide [1,3-5].

There are numerous reports on the effect of physical properties of the substrate on enzymatic hydrolysis and chemical catalytic conversion, with an understanding that increasing the surface-to-mass ratio is an effective way to improve enzymatic saccharification [6]. However, related information has been notably absent for microbial cellulose conversions. In a 1990 study, Weimer and coworkers [7] have investigated the properties of fine cellulose particles and their effect on fermentation rates by a mixed ruminal consortium. For type I celluloses, crystallinity had little effect on fermentation rates whereas there was a strong positive correlation with gross specific area. The authors acknowledged the importance of determining the accessible surface area for microbial attachment and the challenges in estimating this parameter as the substrate topography becomes more complex. As pointed out by Barakat *et al.*[8], cost-effective strategies for substrate modifications, especially mechanical reduction of particle sizes and chemical pretreatments, present serious challenges for biorefineries aimed at lignocellulose conversion. This is also relevant to consolidated bioprocessing, an approach proposed to convert cellulosic feedstock to biofuels [9], utilizing microorganisms to break down and ferment cellulosic substrates in a single process step without added enzymes. Although it is foreseen that feedstock availability will be a major consideration for the adoption of such second-generation biofuels [10], there is also a need to improve reactor performance through organism or substrate selection and engineering, through advancements in reactor process technology, and an overall better understanding of cell-substrate interactions.

It has been shown that *C. thermocellum* biofilms can achieve near-complete substrate hydrolysis in the absence of suspended cell populations [1], suggesting that cellulose conversion is primarily a surface phenomenon (i.e., occurs in the biofilm layer). Therefore, delineating their physiology and performance limitations should contribute to discerning their involvement in cellulosic-carbon flow in reactors and nature. Only a few studies have analyzed cellulose-degrading biofilms [11-13], especially the growth dynamics and carbon utilization by biofilms in the absence of a suspended cell population; the presence of which may interfere with collection of data to describe conversion kinetics specific to the surface-attached population. Continuous-flowcell reactors provide a suitable method for direct observations (e.g., with scanning confocal laser microscopy) of the structure of cellulolytic biofilms, and sampling of the aqueous off-stream provides useful information about biofilm metabolism. However, direct sampling from the reactor for time-resolved performance analysis is not desirable as it may interfere with microbial activity. Also due to the complex, fibrous nature of the cellulosic matrix, microscopy remains mostly descriptive. Therefore, new techniques that analyze cellulose conversion in real time are needed. In batch fermenters, analysis of total gas production measured directly at normal pressure or through displacement, as well as inferred from pressure changes in a closed reactor were validated as potential methods to describe the kinetics of microbial digestion of forage feedstock by ruminal organisms in vitro [14,15]. More recently, online measurement of carbon dioxide off-gas production by Avicel-grown *C. thermocellum* was found to be a robust technique for screening purposes [16]. Although such techniques have been accepted for batch conversions, to our knowledge, they have not been used as metabolic indicators for the study of microbial cellulose conversion in continuous-flow reactors by the predominant activity of cellulolytic biofilms.

In this study we measured the real-time production of CO_2_ during fermentation of pure cellulose sheets by *C. thermocellum* biofilms in continuous-flow reactors. The objective was to use this data in conjunction with imaging and sessile-to-planktonic cell yields to learn more about biofilm colonization and the relationship between accessible substrate surface area and cellulose conversion kinetics, as well as to determine the value of on-line gas measurements as an indicator of microbial activity and substrate digestibility.

## Results

**Figure 1:**
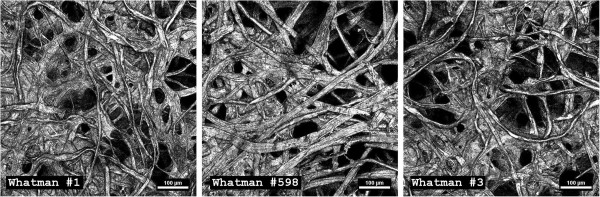
**Substrate heterogeneity.** Micrographs of Whatman filter paper showing highly heterogeneous fiber distribution, pores and channels, which make quantitative differentiation of inner structure properties and of cellulose area available for microbial adhesion not possible. Images taken with confocal reflection microscopy of paper chads sputter-coated with gold. In the current study we refer to biofilm ‘real-estate’ as the portion of the cellulose surface that is accessible to microbial adhesion.

**Figure 2:**
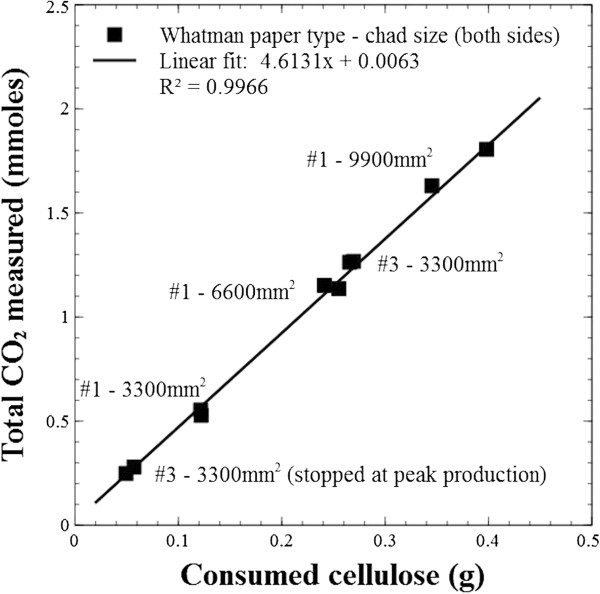
**Correlation between cellulose mass and CO**_**2 **_**production.** A strong positive linear correlation was found between the total carbon dioxide measured and the mass of consumed cellulose. Slope p = 0.000, constant p = 0.792.

A novel configuration of confocal microscopy to enable reflective imaging revealed the highly heterogeneous distribution of fibers and channels in Whatman filter paper # 1, #3, and #598 (Figure 1). Chads of known dimensions were cut out of Whatman paper sheets and incubated under continuous-flow in the presence of *Clostridium thermocellum*. Under steady growth conditions, the production of carbon dioxide gas was compared to the end-point consumption (cca. 92% mass reduction) of cellulose paper chads for Whatman #1 of three different sizes. The relation was also tested for Whatman #3 with experiments stopped either at peak gas production (cca. 18% mass reduction) or at the end-point. A strong positive linear correlation (R^2^ = 0.997) with a zero intercept was found between total gas measured and consumed cellulose for all scenarios tested (Figure 2).

**Figure 3:**
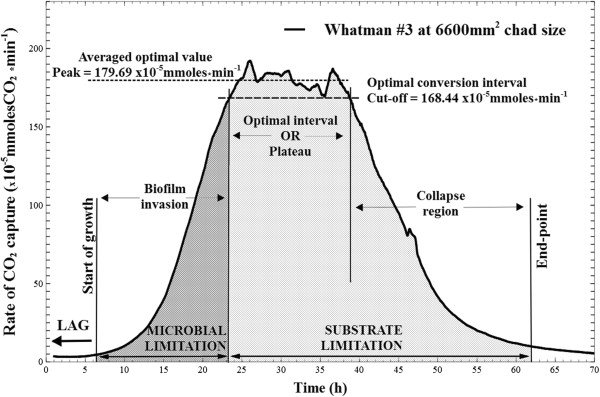
**Model CO**_**2 **_**profile.** Typical measurement of changes in carbon dioxide production rates during microbial conversion of a Whatman #3 paper chad cut at 6600 mm^2^ footprint area (value accounts for both sides of the paper). The working definitions established and used throughout this paper are labeled and delineated: the lag phase from the moment of inoculation varies between seven to nine hours; start of growth is considered at 5 × 10^-5^ mmoles/min carbon dioxide rate and the fermentation end-point at 10 × 10^-5^ mmoles/min; the initial growth period is termed the ‘biofilm invasion’ region, which ends when near-all of accessible surface area (i.e., the ‘real estate’) of the paper has been colonized; it is followed by a ‘plateau region’ of stable, and maximal carbon dioxide output where biofilms advance through the depth of the paper chads (new surfaces become available at the same rate that colonized surfaces are depleted); the last ‘degradation’ region is characterized by rapid loss of available surface and chad integrity.

A typical profile of changes in the rate of CO_2_ production during fermentation cycles is presented in Figure 3. A lag phase of seven to nine hours (data not shown here) preceded the onset of significant reactor activity (5 × 10^-5^ mmolesCO_2_ · min^-1^), followed by a short exponential, then linear rate increase which stabilized in the ‘plateau’ region. Due to small variations in peak output, the optimal conversion interval (i.e., the ‘plateau’) was delineated to include the top 10% recorded values (see Figure 3). The onset of sudden decrease in the CO_2_ production rates (i.e., the ‘collapse’ region) was accompanied by structural disruption of the remaining substrate, where thinned-out chads broke into pieces that accumulated at the bottom of the reactor. A fermentation end-point cut-off value was chosen at CO_2_ production of 10 × 10^-5^ mmolesCO_2_ · min^-1^, where further substrate conversion became slow.

**Figure 4:**
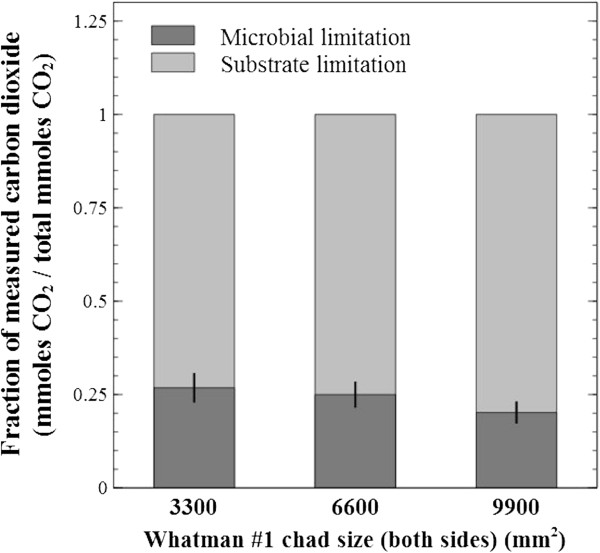
**CO2 production during microbial and substrate limitation zones.** Through integration of the measured rate of carbon dioxide over time, the amount of gas produced during each limitation zone (see Figure 3) was calculated and reported as a fraction of total CO_2_ produced. The substrate limitation zone dominates the overall metabolic activity output by up to 81% of total, a trend observed for all other substrate types tested. During this period, surface accessibility, rather than microbial colonization rate, is the major limiting factor.

As seen in Figure 3, a ‘microbial’ and a ‘substrate’ limitation zone were defined as the time region where cellulose conversion was controlled by these different factors, and the corresponding total CO_2_ produced was calculated by the integration of rates. The fraction of CO_2_ produced during the first zone was calculated to range between 0.20 to 0.27 of total for Whatman #1 of different sizes (Figure 4); and for all other paper types, similar ratios were observed, going as low as 0.17 of the total.

**Figure 5:**
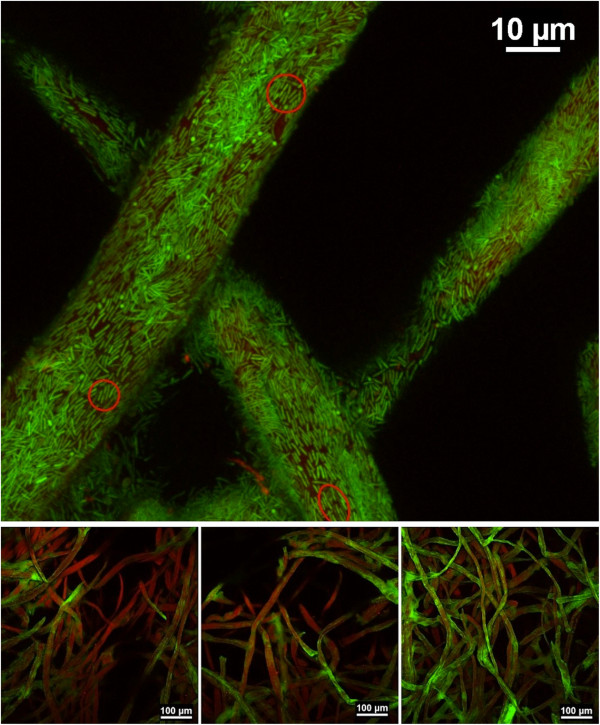
**Biofilm development during microbial limitation.** Confocal scanning micrographs of *Clostridium thermocellum* biofilms on Whatman #3 filter paper chads taken midway during the ‘biofilm invasion’ region. Low magnification images (below) show varying degrees of biofilm colonization (green) on cellulose fibers (red); where it is common to observe surface fibers with little to no biofilm growth. High magnification imaging (above) of selected heavily populated fibers shows the typical cell monolayer biofilms of this species, with cells closely lining the substratum. Dividing cells (red circles) and occasional small spore-forming cells (green dots) are observed at the surface. Cells stained with SYTO 9 (green); cellulose fibers stained with WGA-TRITC (red).

**Figure 6:**
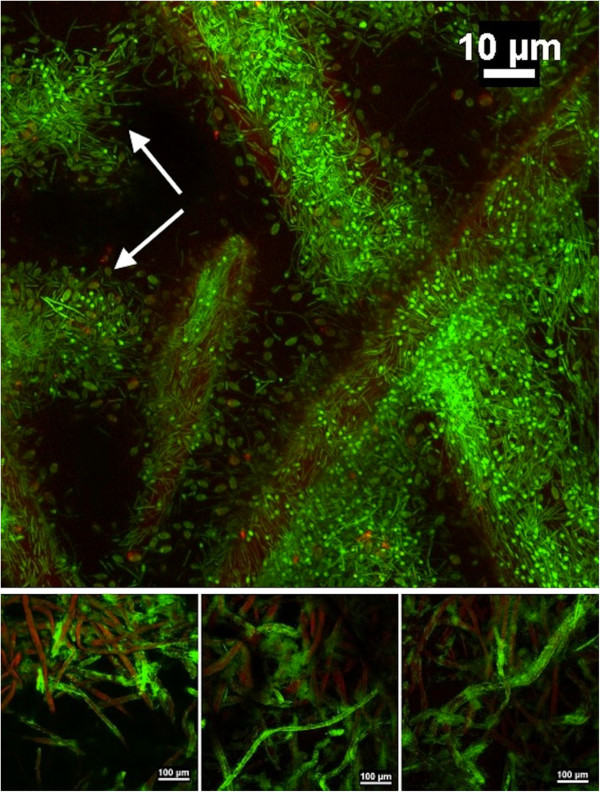
**Biofilm development during substrate limitation.** Confocal scanning micrographs of *C. thermocellum* biofilms on Whatman #3 filter paper chads, taken approximately halfway through the ‘plateau’ (see figure 3) region. Low magnification images (below) show heavily populated fibers, some with very high cell-density biofilms, which were consistently observed throughout. Fibers with lesser colonization were observed typically deeper into the paper structure. High magnification imaging (above) of an extremely dense region shows the formation of numerous terminal sporangia, attached to the substratum at the non-sporulated end. Normal cells are still observed to line the surface of the same fibers. The regions indicated with white arrows show near-complete collapse of the cellulose fibers. Cells stained with SYTO 9 (green); cellulose fibers stained with WGA-TRITC (red).

Microscopic observations of the degree of biofilm colonization were made midway through the ‘biofilm invasion’ and the ‘plateau’ regions (as labelled in Figure 3). The first region was characterized by incomplete biofilm coverage of the superficial cellulose fibers, ranging from sparsely distributed sessile cells to patches of uniform coverage (Figure 5, low magnification). Adherent cells were observed on fibers as deep as 20 to 30 microns from the surface. At higher resolution the typical *C. thermocellum* biofilms were revealed, with a single layer of cells lining the substratum. Frequently, cells appeared aligned with each other, which was indicative of division at the interface along their longitudinal axis (Figure 5, high magnification). By contrast, imaging during the plateau region consistently revealed heavy fiber colonization by dense biofilms with significant penetration into deeper layers (Figure 6, low magnification). It was not uncommon to observe biofilm areas with a remarkably high count of spore-forming cells (Figure 6, high magnification).

**Figure 7:**
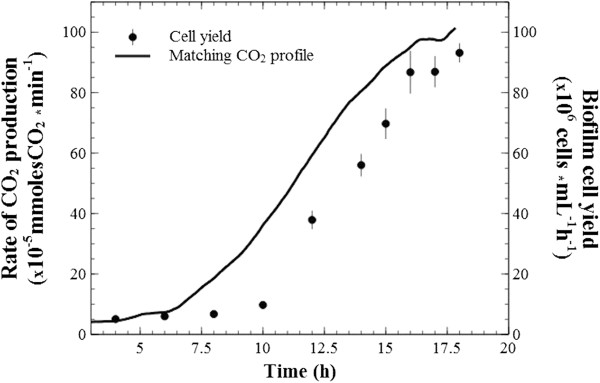
**Biofilm to planktonic cell yield.** Numbers of cells in reactor effluent as indication of biofilm-to-planktonic cell yield and corresponding (matching) CO_2_ production rate during the microbial limitation period and a few hours into plateau.

The rate of cell elution from biofilms growing on Whatman #3 chads was measured in parallel with on-line rates of CO_2_ production (Figure 7). Cell elution initially lagged behind then increased to match higher culture activity, and appeared to stabilize at peak CO_2_ production rates.

**Figure 8:**
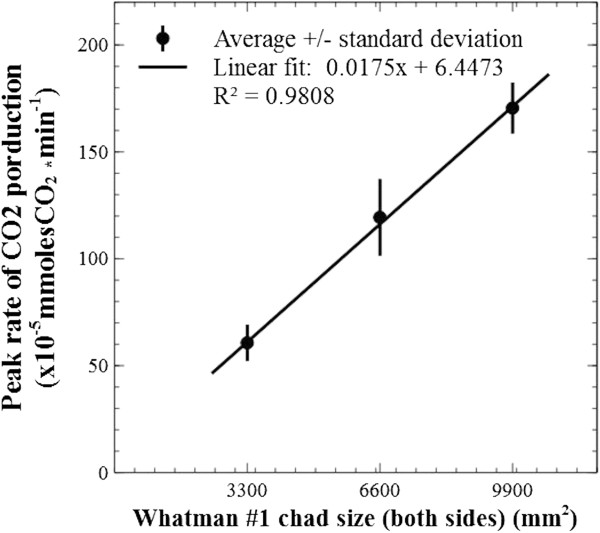
**Correlation between surface footprint area and CO**_**2 **_**production.** A strong positive linear correlation was observed between the measured peak rate of carbon dioxide production and the initial size of Whatman #1 sheets. This shows initial biofilm invasion proceeded to the extent at which available ‘real estate’ was provided, and that surface area was not lost before this point was reached (mainly due to the flat shape of the cellulosic chads; the sheet with the least thickness was chosen for this test). It also emphasizes the utility of such measurements for comparing the degree of bacterium-accessible surface area of complex and heterogeneous substrates. Slope p = 0.000, constant p = 0.560.

**Figure 9:**
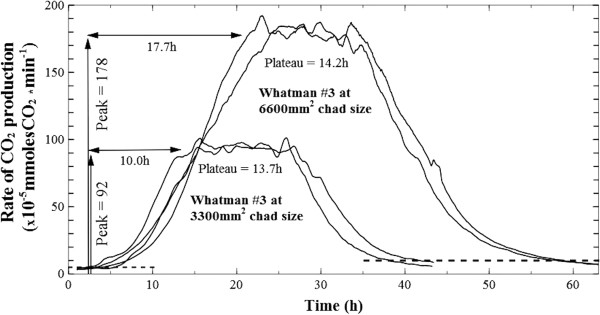
**Rates of CO**_**2 **_**production during fermentation of Whatman #3 paper chads of 3300 mm**^**2 **^**and 6600 mm**^**2 **^**footprint areas.** Peak rate doubled, microbial limitation increased and plateau length remained at comparable values for chads with identical thickness but different size.

To test the relation between peak rates of CO_2_ production and substrate ‘real-estate’ availability, carbon dioxide profiles were recorded for paper chads with areal dimensions increased by a factor of one, two and three (i.e., the paper sheets were cut at larger dimensions, which would also translate to a proportional increase in internal attachment surface). A strong positive linear correlation (R^2^ = 0.981) was observed between peak CO_2_ rates and chad sizes (Figure 8), with a near-zero intercept strongly suggesting that CO_2_ production is directly proportional to the accessible surface area. The relationship was first tested for the thinnest paper type, Whatman #1, and later also observed for the thicker sheets (see Figure 9, Whatman #3).

**Figure 10:**
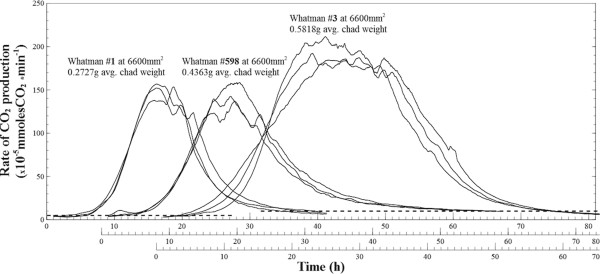
**CO**_**2 **_**production on different substrate types.** Changes in the rates of carbon dioxide production were compared for same-sized (6600 mm^2^) chads of Whatman paper #1, #598 and #3. Plots are staggered on the x-axis and the corresponding mean mass of each chad type labeled; the black dotted lines denote the cut-off points set for the start of growth and the end-point fermentation.

**Figure 11:**
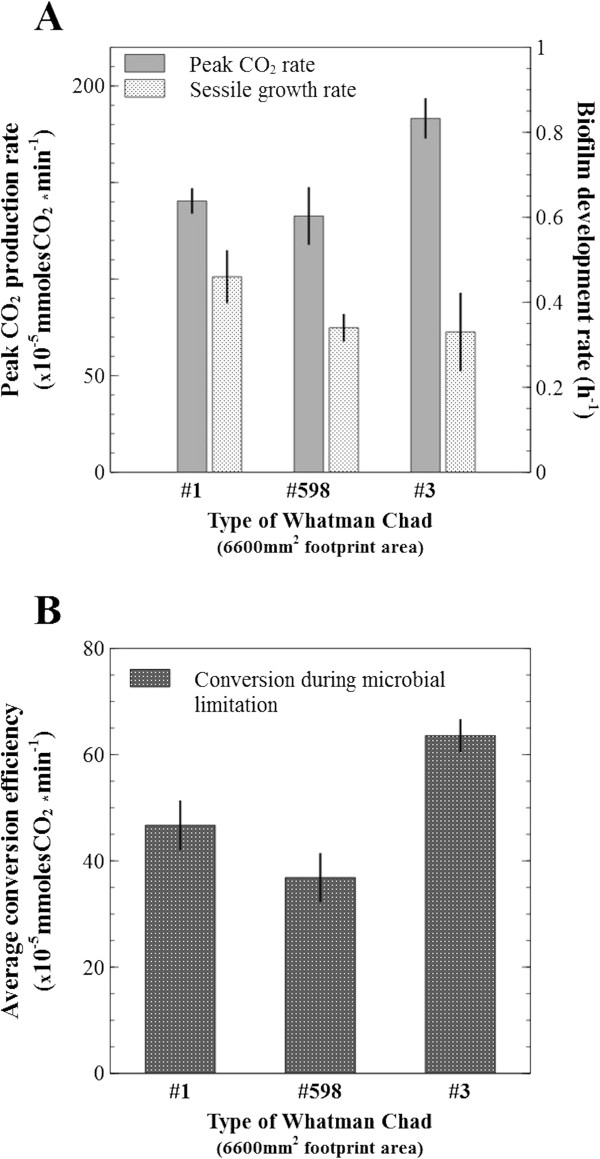
**Conversion efficiency during microbial limitation. (A)**. Measurement of CO_2_ production as an indication of biofilm development rate, and the peak activity on three cellulosic substrates with equal size, but different mass. **(B)**. Comparison of average conversion efficiency during the initial microbial limitation zone. A doubling in rates of gas production is understood as a doubling of sessile population size, and peak rates are strongly influenced by availability of attachment surface area. The combination of these factors reveals the average conversion efficiency during microbial limitation and it becomes an indicator of biofilm colonization potential (higher is better). Error bars show standard deviation.

Gas production profiles of equally-sized chads (i.e., 6600 mm^2^) for all sheet types were also compared (Figure 10). Whatman #1 and #598 achieved similar peak rates (p = 0.437) while Whatman #3 was consistently higher (compared to #1, p = 0.003) (Figure 11A). To verify that higher gas rates were related to surface exposure area, Whatman #3 chads of half the size were digested in identical conditions (Figure 9). The response in peak activity was proportional, and in general the CO_2_ profiles have mirrored the larger sheets, in terms of rate changes and plateau duration.

A doubling in gas production rates (i.e., from 50 to 100 × 10^-5^ mmolesCO_2_ · min^-1^, Figure 10) during the initial linear increase phase was interpreted as a doubling of sessile population size. Therefore, the biofilm development rate was calculated (Figure 11A) to be similar between Whatman #598 and #3, at 0.34 h^-1^ and 0.33 h^-1^, (p = 0.852), and faster, 0.46 h^-1^, on Whatman #1 (compared to #598, p = 0.033; and to #3, p = 0.094).

As substrate mass under ‘microbial limitation’ was considered in excess, the average efficiency for converting cellulose to metabolites in this region (measured in mmoles CO_2_ · min^-1^) was highest for Whatman #3, significantly slower for Whatman #1 (p = 0.005), and further reduced in Whatman #598 (p = 0.054) (Figure 11B).

**Figure 12:**
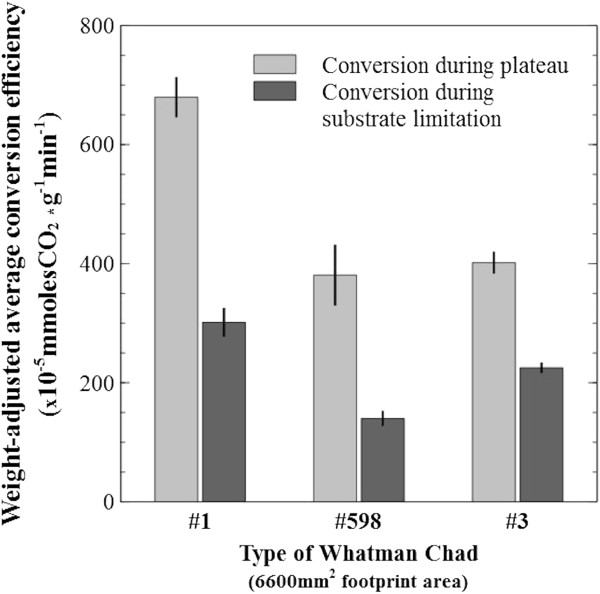
**Conversion efficiency during substrate limitation.** Comparison of substrate digestibility calculated as mmoles (CO_2_) · g^-1^ · min^-1^ during plateau and throughout the substrate limitation zone. The mass of equally sized paper chads is adjusted to account for the portion consumed during microbial limitation (based on fractional CO_2_ production). Whatman #598 appears to be the least degradable paper type.

Substrate digestibility under the plateau and under the entire ‘substrate limitation’ zone was calculated in mmolesCO_2_ · g^-1^ · min^-1^ (normalized to mass, which was considered a limiting factor for these regions) and compared between all paper types (Figure 12). Digestibility during the plateau was significantly better for Whatman #1 (p < 0.001), while #598 was the overall worst performer.

**Figure 13:**
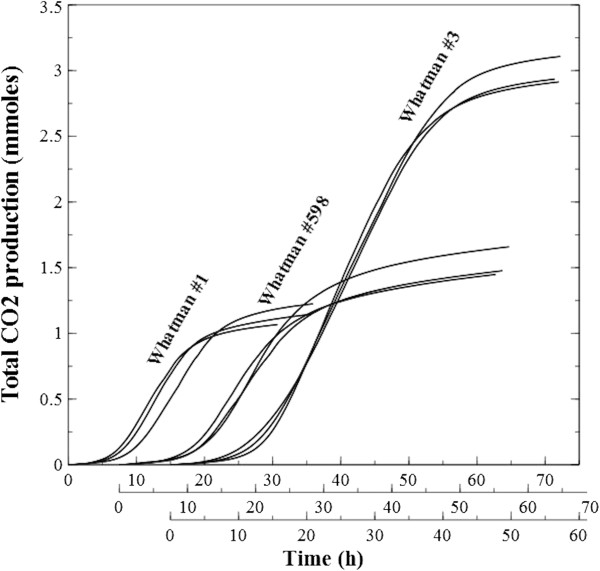
**Cumulative CO**_**2 **_**production.** Plotting of cumulative carbon dioxide production from start of activity to the selected end-point cut-off for equally-sized Whatman paper chads. The sigmoid-shaped curves are in agreement with previously described logistic (or Richards) models of solid cellulose conversion through microbial activity.

Cummulative plotting of carbon dioxide produced from the start to the end-point fermentation of the equally-sized cellulose chads revealed a general sigmoid shape (Figure 13) with an accelerated, steady and then decelerated metabolite production.

## Discussion

The leading objectives of this study were to advance our apprehension of cellulolytic biofilm formation and to assess the importance of cellulosic-surface accessibility to biofilm activity and conversion kinetics. Studies on pure cellulosic substrates are typically performed with Avicel particles, which have a smooth appearance and very large specific surface area. For physically complex substrates, determining the accessible surface for biofilm colonization (i.e., the biofilm ‘real-estate’) becomes a daunting task. Area evaluations of surfaces with micro-scale roughness are typically made by calculating mean height deviations. For example, Whatman #1 fibril roughness has been estimated at approximately 450 nm (root mean square of a 10 × 10 μm^2^ area) [17]; however, high heterogeneity in fiber distribution and overall porous appearance (Figure 1), make an accurate interpretation of three-dimensional topography difficult. Adhesion of cellulolytic bacteria (of 2 × 0.5 μm average size) contributes to the uncertainty, as the degree of depth penetration is unknown. Because adherent *C. thermocellum* cells are known to be primary hydrolyzers and consumers of solid substrates, cellulose conversion by this, and related bacteria is proposed to be primarily a surface reaction; therefore reliable data on the extent of biofilm growth and surface accessibility is important. It was consequently necessary for our research to develop a method to track whole-biofilm development and overall reactor activity in real time.

Carbon dioxide is produced stoichiometrically with the two major metabolites of cellulose conversion (acetic acid and ethanol) by *C. thermocellum*. Under the given conditions, CO_2_ measurements proved to be a reliable indicator of cellulose conversion throughout the fermentation cycles (Figure 2). We acknowledge that the gas-produced to cellulose proportions are likely to change when substrates with different chemical compositions are compared . For these situations we advise online gas measurements to be used as a metric of metabolite output (i.e., ethanol and acetic acid).

Continuous-flow reactors were operated at 1 h^-1^ dilution rate, which is at minimum threefold higher than previously reported specific growth rates for *C. thermocellum*[5,16], therefore free-floating planktonic populations were continuously washed out of the reactor. As the temperature of the reactor effluent sharply dropped from the 60°C optimum inside the reactor to 4°C in the effluent collector, all effluent measurements were interpreted as a product of the cellulose-adherent sessile population inside the reactor. At steady operating conditions, the increase or decrease in the rate of CO_2_ production in the reactor was interpreted to emulate changes in the number of active adherent cells; however, it is acknowledge that this may not be the case for substrates that become less reactive. During ‘biofilm invasion’, bacteria penetrate and colonize exposed fibers in the porous matrix. When all accessible attachment surfaces are occupied (i.e., biofilm real-estate is maximized) a peak rate in CO_2_ output is achieved. At plateau, it is hypothesized that the active sessile population remains constant at maximum coverage and the cellulose sheets are reduced in thickness. Once the chad structure cannot be maintained (as corroborated by visual observations), the CO_2_ production rate sharply drops during the ‘collapse’ region where substrate surface and, consequently, sessile cells are lost.

Microscopic observations substantiate this model of biofilm penetration from the surface to the interior. Cell density on fibers increases through division of adherent cells. This is coupled with continuous detachment of cells, a fraction of which would re-attach at different sites. However, since these biofilms are primarily cell monolayers, the total sessile mass is controlled by substrate-surface availability. Conceptually, biofilm-to-planktonic cell yield occurs through cell division and subsequent release from the cellulosic surface, and through bulk dissolution of biofilms that have consumed their support substratum. As such, bulk dissolution is not expected to be significant during early stages of fermentation, and as shown in Figure 7, cell elution initially lagged behind culture activity. Subsequently, elution rate emulated the CO_2_ profile, in agreement with previous data [3], which confirms that biofilm cell yield can be expected to match surface colonization.

We have previously reported sporulation to occur during normal growth conditions on solid substrates [1,18]. A characteristic in the present study that was in agreement with our earlier observations was the high density of cells competing for limited cellulosic surface. It appears that sporulation in this case is a stress response mechanism, with yet unknown physiologic triggers, to the lack of available substratum. Possible underlying reasons include: i) poor access to the substrate leads to carbon starvation, in which case sporulation falls within the classical survival theory; ii) cells have taken up sufficient cellulosic oligomers and are reverting to the resistant spore-form for energy conservation, in which case sporulation can be seen as a pro-active cyclic event; iii) the bacterium has a functional species-specific auto-induced quorum sensing mechanism, with the property of regulating gene expression in response to cell population density, in which case sporulation becomes a tool of species proliferation by preparing the cells for translocation to new cellulosic substrates. For the latter proposition, it is interesting to note that spores of various thermophilic bacilli, unrelated to *C. thermocellum*, were found to have higher propensity for surface attachment than vegetative cells [19], with some that may establish monolayered biofilms [20].

The results of Figures 8 and 9 provide a strong argument that, for a given substrate, the accessible surface area defines the maximum rate of cellulose conversion by providing an upper limit to the extent at which a sessile population of thin biofilms can grow. It is important to note the cellulose sheets’ footprint area was not affected before peak production; a benefit of utilizing substrates with flat-sheet shape (and almost pure cellulose), where loss of mass was not readily translated in loss of area. However, it is expected that significant increases in the substrates’ surface to mass ratio will lead to the loss of footprint area before the true biofilm real-estate potential could be reached. In terms of rate kinetics is appears that microbial cellulose conversion is initially limited by microbial colonization and sprawling, while substrate mass is in excess (Figure 3, the ‘microbial limitation zone’), and the rate will increase until all available substrate has been colonized.

As seen in Figures 10 and 11A, the peak rate of CO_2_ production was notably higher for Whatman #3 even though the chads were cut to identical size, which is suggestive of a different surface topography and a better accessible internal structure. As discussed earlier, and shown in Figure 1, accurate assessment of available surface for microbial attachment on these matrices, through direct measurement, is difficult to achieve. However, because of the linear correlation shown in Figure 2 between the total CO_2_ produced and the mass of consumed cellulose, it is reasonable to assume that CO_2_ profiles provide a means to compare different substrates.

An analogy can be made between biofilm development on cellulosic substrates and catalyst addition to an enzymatic reaction, where the rate increase in activity is telling of the rate at which catalyst was added, and the maximum achievable activity value is telling of the total quantity of catalyst supplied. Along these lines, biofilm colonization potential can be inferred by calculating the average conversion efficiency of cellulose during the microbial limitation zone (Figure 11B). It appeared that Whatman #3 provided the best overall accessible surface for microbial colonization and hydrolysis. Interestingly, although Whatman #1 and #598 provided comparable overall surface real-estate (as compared by peak activity rates, Figure 11A), the former had the better colonization potential as shown by the faster biofilm development rate.

In the plateau region, it is proposed that sessile cells have colonized all available substrate and the chads’ footprint area was maintained while biofilms progress through the thickness of the sheet (see Figure 9). Substrate digestibility throughout the depth profile of the sheet can be compared as the weight-adjusted conversion efficiency to CO_2_ gas (Figure 12). Whatman #1, which was estimated to be the densest, showed the highest values. This could be due to being better at staying intact, and thus maintaining its footprint area. Interestingly, when substrate digestibility to end-point fermentation was compared for the entire substrate limitation zone, the worst performer was Whatman #598, the least dense paper. Further evidence that this paper type is the most recalcitrant of the 3 types tested, is the elongated tail in the collapse region (Figure 10), also reflected by the upper asymptotes of Figure 13.

For all paper types, cumulative CO_2_ production plotted over time (Figure 13) had a characteristic sigmoid shape. Similar profiles are reported in the literature for total off-gas production during fermentation of pure cellulose by mixed ruminal consortia [21] and for carbon dioxide production by *Clostridium thermocellum* on Avicel crystals in batch systems [22]. Both reports propose a logistic (or Richards) model for substrate reduction with rate kinetics of first order with respect to cell mass, first order with respect to substrate concentration and second order overall. The Schofield group [21] used gas measurements to describe kinetics, while Holwerda and collaborators [22] preferred the quantification of biosynthate and substrate concentration. Both models propose a duality of conversion rate limitations where neither cell mass nor substrate concentration can be assumed in excess. Our results are in agreement for the duality of limiting factors, and the cumulative CO_2_ production profiles verify the applicability of our approach; however, we propose that a period of microbial limitation (controlled by species or environment-specific properties) always precede a period of substrate limitation, with the overlap between them being minimal when compared to total reaction time. As such, substrate is in excess until peak activity (as measured in CO_2_ production in this case) is reached, followed by the excess of microbial mass while substrate becomes limiting. The fact that there is a sustained yield of suspended cells at relatively high numbers (typically ~10^7^ – 10^8^ cells · ml^-1^ effluent) from the biofilms for the duration of the plateau period supports this assertion. Considering bacterial cellulose hydrolysis is largely a surface reaction, it should be beneficial for future kinetic models to recognize cellular mass as a dynamic body of two distinct populations (planktonic and sessile) with different propensities, and potentially roles, in cellulose conversion.

## Conclusions

Understanding the changes in rates of CO_2_ production can be used to estimate the surface available for microbial attachment on cellulosic substrates, to indirectly estimate the rate of sessile growth and to provide a relative comparison of substrate digestibility under the two distinct limitation zones. As it is seen with our examples, increasing surface availability extends the period of microbial limitation (Figure 9), an aspect that may have to be considered when utilizing microbes with low specific growth, or more appropriately, specific biofilm development rates. Maintaining peak activity rate is generally achieved by sustaining surface footprint while the mass is reduced, an aspect that favours flat or sheet-type particles over spherical shapes. Substrates with higher densities appear to have an innate advantage towards footprint maintenance. In an applied sense – as feedstock modifications (e.g., increasing surface exposure by mechanical reduction in size) are generally associated with increasing processing costs, it is important to understand the tools for comparative analysis and to achieve an acceptable balance between microbial and substrate limitations for effective conversions. The approach described here may be useful in this regard, even though pure cellulosic substrates were used. It will be worthwhile testing its applicability on lignocellulosic feedstock.

## Methods

### Bacterial strains and chemicals

*Clostridium thermocellum*, strain 27405, donated by Dr. Paul Weimer (University of Wisconsin), was maintained in the laboratory over many generations in RM medium [23] supplemented with Avicel PH-101. All reagent-grade chemicals were purchased from Sigma-Aldrich Co. (MO, United States) or VWR International (PA, United States) while fluorescent stains were acquired from Life Technologies Inc. (ON, Canada). Compressed gases were purchased from Linde Canada Ltd. (ON, Canada). Ultra-pure water used in medium preparation and assays was processed with a Milli-Q Gradient system from EMD Millipore (ON, Canada).

### Culture media

Medium composition and preparation used for batch cultivations were previously described [1]. The continuous-flow reactor (CFR) medium used in this study was an evolution of our MC medium with the addition of 1 g to the liter of MOPS (3-(N-morpholino) propanesulfonic acid) hemisodium salt for extra buffering capacity, while preparation was not changed [1].

### Continuous-flow reactors and substrate preparation

Cellulose filter paper (Whatman #1, #598 or #3, with minimum 98% alpha cellulose) was packed into 20 mL reactors, which consisted of modified glass crimp-seal vials with inlet and outlet ports. The reactors were sealed with rubber stoppers (Bellco Glass Inc., NJ, United States), and Masterflex Norprene and Tygon tubing (Cole Palmer Inc., Canada) made the connection with the medium container at the inlet port and a peristaltic pump (model 205S, Watson Marlow, Cornwall, England) at the outlet port, respectively (Additional file 1: Figure S1). The reactors were immersed in a 60°C sealed water bath that was continuously purged with nitrogen gas to provide uniform water heating. The reactor effluent was pumped at 20 mL/h through a carbon dioxide evolution measurement system (CEMS) described elsewhere [24] into a sterile collection vessel maintained at 4°C. To prevent contamination, the assembled tubing and reactors were sterilized with 200 mL of 1% sodium hypochlorite for 2 hours at 100 mL/h flow rate, and then washed overnight with sterile water at normal flow before switching and purging with freshly prepared medium for 3 hours. Reactors were inoculated directly through the rubber seal (25% of reactor volume) with fridge-stored stock cultures of *Clostridium thermocellum*, while medium flow was halted for 2 hours to allow cell acclimation. Medium flow was then resumed at normal speed and the inoculum residues were flushed into a separate waste container for the initial 3 hours.

Whatman paper sheets were cut to the nearest 0.2 mm into chads of 55 mm length and 30 mm, 60 mm or 90 mm width, for a total footprint area (i.e., both sides) of 3300 mm^2^, 6600 mm^2^ and 9900 mm^2^, respectively. They were dried at 120°C for 3 hours, weighed and carefully packed into reactors, such that the entire surface of the chads was fully exposed. At end-point fermentation, residual cellulose was collected on 934-AH Borisilicate glass fiber filters (Sterlitech Corporation, WA, United States), thoroughly washed with milli-Q water, dried and weighed. Manufacturer specifications indicate the typical thickness (in μm) of Whatman #1, #598 and #3 paper sheets at 180, 320 and 390, respectively. Based on dry weight, the calculated densities (in g · cm^-3^) are 0.4591, 0.4132 and 0.4521, in the same order.

### Imaging

Cultures were stained, at the specified times, *in-situ* by injection of a one milliliter mixture of the green nucleic acid stain, SYTO 9 (15 μM), and the carbohydrate-binding lectin, WGA-TRITC (15 μg/mL). Flow was arrested for 15 minutes to allow stain penetration, and the filter paper was then carefully removed and cut into four quadrants. Diagonal quadrants were placed on microscope slides with opposite sides facing upward, then saturated with mounting oil and protected with a coverslip. Confocal laser scanning images were acquired with a Nikon Eclipse 80i-C1 microscope (Nikon Instruments Inc., Canada) using the 20×/0.75 NA oil immersion Plan Fluor and the 60×/1.4 NA oil immersion Plan Apo objectives. Sample volumes were scanned at random positions with 0.62 μm/px density and 1.00 μm z-step size under lower magnification and with 0.21 μm/px density and 0.30 μm z-step size for the higher magnification. The EZ-C1 software package was used for acquisition and the NIS-Elements software package (Nikon Instruments Inc., Canada) for image processing.

Fresh, unprocessed Whatman paper chads of all types were gold sputter sprayed with a Desk IV (Denton Vacuum, NJ, United States) for 60 seconds (at 110 mTorr, 32 mAmp and 60% sputter setpoint). Gold-sputtered chads were imaged with a 20×/0.5 Plan Fluor objective under ‘confocal reflection’ mode using the same microscope with modified configuration. The microscope scanhead dichroic was changed to a beam splitter (BS 20/80) and the 488 nm Argon laser was used to illuminate the samples. Reflected light was captured with a photomultiplier tube detector that had all filter cubes removed. Images were acquired at 0.62 μm/px density and 1.1 μm z-step size and processed with the same software.

### Cell counts

Reactor effluent was collected at 4°C continuously, in hourly aliquots, from the beginning of culture activity through the peak rate region as determined by CO_2_ production (see below). For each aliquot, one milliliter dilutions were prepared, as needed, in saline solution mixed with 4',6-diamidino-2-phenylindole (DAPI, at 12.5 μg/mL final concentration) nucleic acid stain. After 20 minutes of dark incubation at room temperature, diluted aliquots were vacuum filtered through Nucleopore polycarbonate membrane filters (25 mm diam, 0.2 μm pore size, Whatman, VWR, Canada), uniformly distributing the sample content over 176.6 mm^2^. Filters were oil-mounted onto microscope slides and fifteen images were taken at random positions for each sample using a Nikon Eclipse 80i-C1 microscope in wide field fluorescence mode. UV illumination and a DAPI filter cube were used with the 20×/0.75 oil immersion Plan Fluor objective, and all images were manually processed with the “cell counter” plug-in of the Image J software.

### Online carbon dioxide measurements

Aqueous effluent from the reactor was passed through the CEMS at 20 mL/h. The device consisted of inner silicone tubing with high gas permeability, which received the effluent liquid, and outer Tygon tubing of large diameter, and very low permeability, that received a carrier gas (i.e., CO_2_-free nitrogen) at 40 mL/min. Dissolved carbon dioxide, produced in the reactor, passed from the effluent liquid through the silicone wall into the annular space of the outer tubing, where it was collected by the carrier stream and passed through an infrared gas analyser (model LI-820 CO_2_ Analyzer, LI-COR Biosciences, ME, United States), which was calibrated against known standards. At constant operating conditions (e.g., pH, temperature), the portion of carbon dioxide transferring into the carrier gas is highly linear (R^2^ = 0.999) with the concentration of dissolved gas in the aqueous feed [24]. The final waste effluent vessel was continuously sparged with nitrogen and passed in real-time through a second analyzer to measure CO_2_ that remained in solution through the CEMS. It was determined that the CEMS consistently captured cca. 65% of total dissolved CO_2_. All data reported in this document represents the CEMS-measured CO_2_ portion. The gas analyzers recorded in the range of 0 to 2000 ppm, with a manufacturer reported measuring noise of less than 1 ppm.

#### Correlation between cellulose utilization and CO_2_ production

In order to test the accuracy of measured CO_2_ production as an indication of cellulose utilization, the residual mass of paper chads of three different sizes (mass) and total CO_2_ production were measured and correlated for either consumption until the end-point of fermentation or until maximum gas output rates were reached.

#### Correlation between accessible substrate surface area and conversion kinetics

Whatman #1 paper was cut into chads of size 3300, 6600 and 9900 mm^2^ and correlated to the CO_2_ data recorded at maximum rate. This peak rate was defined as the average of the top 10% recorded values; this rule was followed in all experiments.

#### Effect of substrate physical properties on conversion kinetics

The three different filter types were cut to all have a total 6600 mm^2^ footprint area, and their respective CO_2_ production measured to determine biofilm development and peak cellulose conversion rates, as well as average conversion efficiency.

### Carbon dioxide data analysis

Gas analyzers recorded CO_2_ concentration (in parts per million), temperature and pressure in the measuring cell at one-minute intervals. Rates of CO_2_ capture (expressed in mmoles · min^-1^) were calculated using the trapezoidal rule:

$$\frac{n{\left(co_{2}\right)}_{i}}{t_{i}-t_{i-1}}=\frac{\mathrm{PQ}}{2\mathrm{RT}}\sum_{i=1}^{i=j}\left(\frac{\chi_{i}}{1-\chi_{i}}+\frac{\chi_{i-1}}{1-\chi_{i-1}}\right)$$

where $n_{{\left(co_{2}\right)}_{i}}$ represent the moles of carbon dioxide at measurement time *t*_*i*_, *P* and *T* the pressure and temperature recorded, *R* the gas constant, *Q* the volumetric flow rate of the carrier gas and *χ*_*i*_ the mole fraction of CO_2_ in the carrier stream at measurement *i*. Average conversion efficiencies were calculated as the total moles of CO_2_ (i.e., integration of rate) captured for a selected time interval.

For graphing purposes, and for an easier visual interpretation, plotted rates of CO_2_ production are represented as one-hour averages of the values calculated at one-minute intervals. A comparison of raw data plotting versus smoothened lines (Additional file 2: Figure S2) shows that shape and inflections are not affected. However, all numerical calculations were made with the original raw data.

### Statistical analysis

Where appropriate, the equality of means between compared parameters was analyzed with an un-paired two-tailed t-test using the Mystat software package. The significance is reported as the p-value; the probability that the observed difference between compared means is due to random chance. Alternatively, 1-p becomes the confidence that the compared means are different. Linear regressions (Figures 2 and 8) were constructed with the ordinary least-square method (OLS) and the regression parameters were tested for significance with a t-test (i.e., whether they are different from zero). A high p-value (over 0.05) indicated that the parameter is not significantly different from zero.

## Competing interests

The authors declare that they have no competing interests.

## Authors’ contributions

AD planned and performed all experiments, wrote the first draft of the manuscript, and was primarily responsible to combine all inputs from the co-authors to finalize the manuscript. GMW initiated the research and participated throughout in the experimentation, as well as writing and finalizing of the manuscript. DGA was involved in the design of experiments, critically revised the manuscript for technical and intellectual content, and gave approval of the final version. SL was involved in initiating the research, critically revised the manuscript for technical and intellectual content, and gave approval of the final version. LL was involved in initiating the research, had frequent inputs on the overall direction of the study, critically revised the manuscript for technical and intellectual content, and gave approval of the final version. All authors read and approved the final manuscript.

## Supplementary Material

Additional file 1: Figure S1 Diagram of continuous-flow reactor systems. Click here for file

Additional file 2: Figure S2 Comparison of raw and smooth data plotting. Click here for file
