# Supplementary figures and images for: Tracking the cellulolytic activity of Clostridium thermocellum biofilms
Source: Biotechnol Biofuels. 2013 Nov 29;6:175. doi: 10.1186/1754-6834-6-175 (PMC4176736; doi:10.1186/1754-6834-6-175)

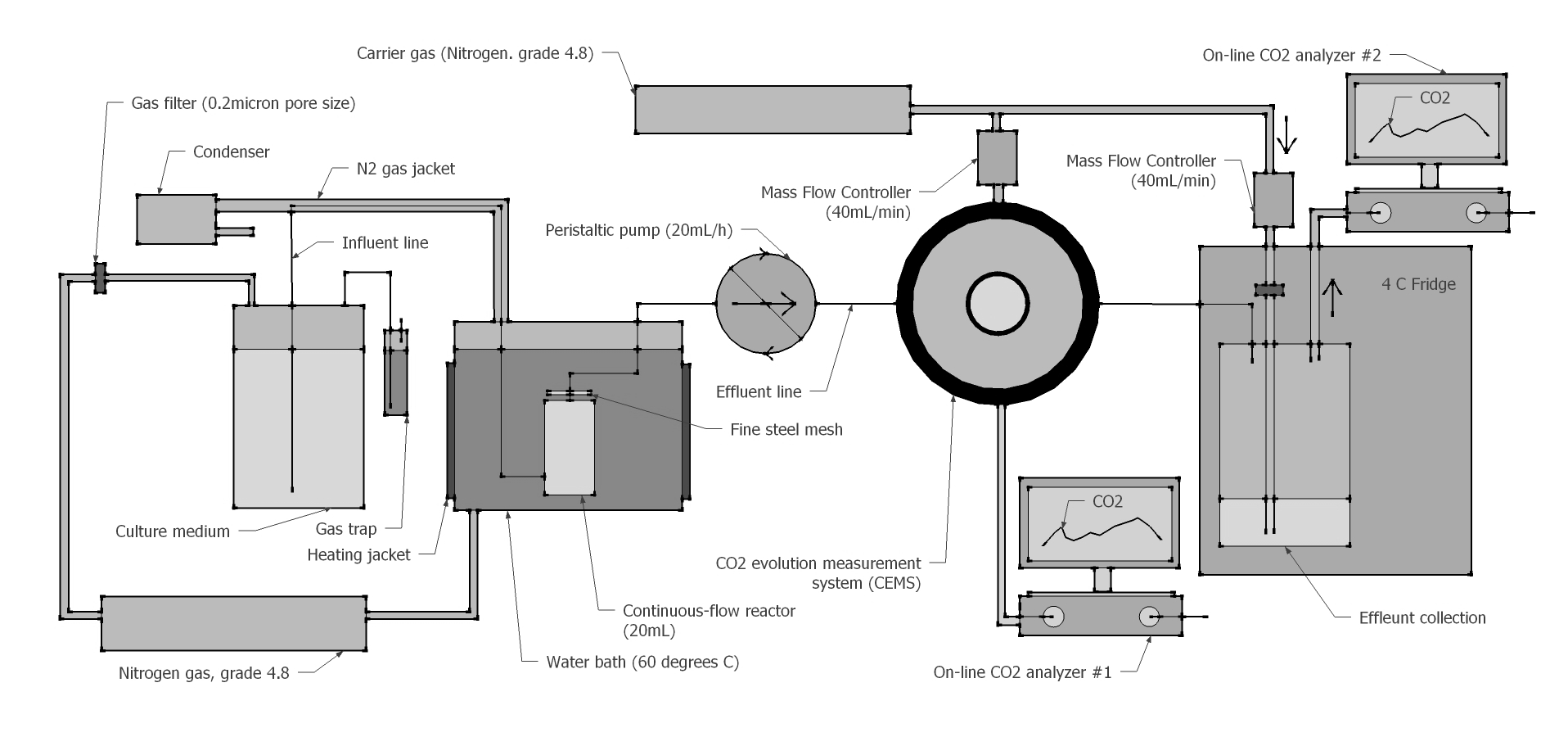

Supplement: Additional file 1: Figure S1 — Diagram of continuous-flow reactor systems. [file 1754-6834-6-175-S1.jpeg]

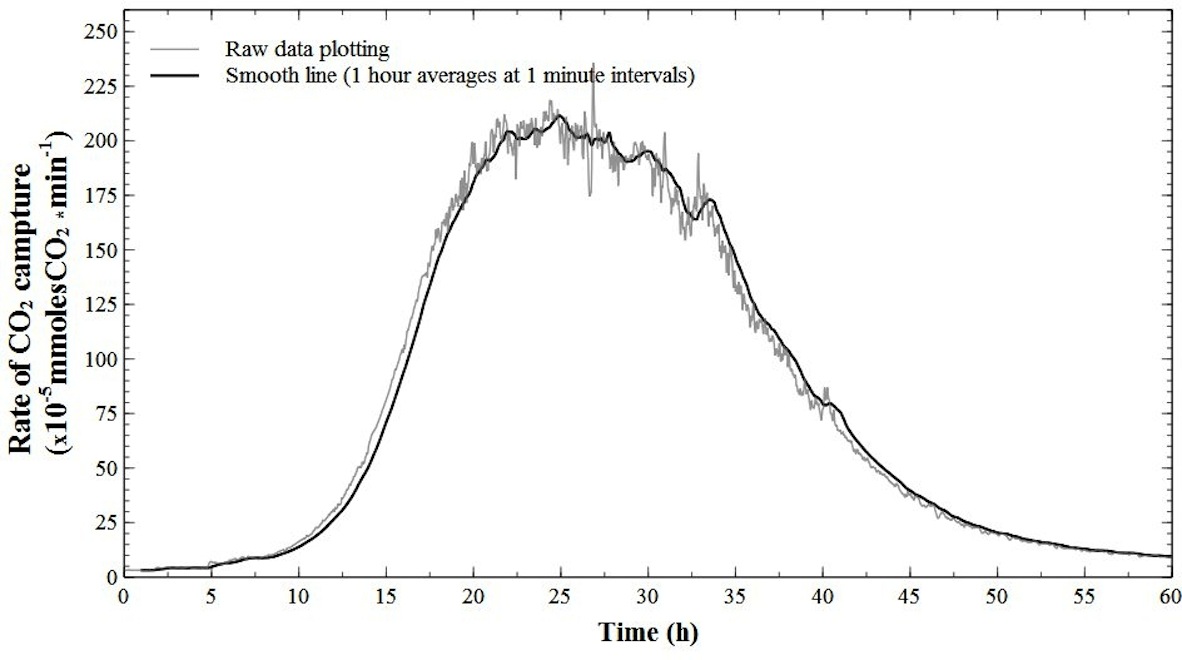

Supplement: Additional file 2: Figure S2 — Comparison of raw and smooth data plotting. [file 1754-6834-6-175-S2.jpeg]
